# SARS‐CoV‐2 Delta‐variant breakthrough infections in nursing home residents at midterm after Comirnaty® COVID‐19 vaccination

**DOI:** 10.1002/jmv.27799

**Published:** 2022-04-27

**Authors:** Ignacio Torres, Juan B. Bellido‐Blasco, Concepción Gimeno, Javier S. Burgos, Eliseo Albert, Raúl Moya‐Malo, Juan Carlos Gascó‐Laborda, Ana Tornero, Josefa Soriano, Noemí Meseguer‐Ferrer, María Martínez‐Serrano, Javier Ortíz‐Rambla, Helena Buj, Noelia Hernández, Salvador Peiró, Dolores Salas, Ramón Limón, Hermelinda Vanaclocha, José Sánchez‐Payá, Javier Díez‐Domingo, Iñaki Comas, Fernando González‐Candelas, David Navarro, the Valencian vaccine research program (ProVaVac) study group

**Affiliations:** ^1^ Microbiology Service, Clinic University Hospital, INCLIVA Health Research Institute Valencia Spain; ^2^ Sección de Epidemiología, Centro de Salud Pública de Castellón Valencia Spain; ^3^ Centro de Investigación Biomédica en Red de Epidemiología y Salud Pública (CIBERESP) Valencia Spain; ^4^ Universitat Jaume I (UJI) Castelló Spain; ^5^ Microbiology Service, Consorcio Hospital General Universitario de Valencia Valencia Spain; ^6^ Department of Microbiology, School of Medicine University of Valencia Valencia Spain; ^7^ General Directorate of Research and Healthcare Supervision, Department of Health Valencia Government Valencia Spain; ^8^ Centro de Salud Carinyena Vila‐Real Castellón Spain; ^9^ Primary Health Directory Consorcio Hospital General Universitario de Valencia Valencia Spain; ^10^ Unidad de Hospitalización Domiciliaria del Departamento de Salud de la Plana Castellón Spain; ^11^ Laboratory Service, Hospital de la Plana Vila‐Real Castellón Spain; ^12^ Foundation for the promotion of Health and Biomedical Research of the Valencian Community (FISABIO) Valencia Spain; ^13^ Department of Health General Directorate of Public Health, Valencia Government Valencia Spain; ^14^ Department of Health General Directorate of Healthcare, Valencian Government Valencia Spain; ^15^ Preventive Medicine Service Alicante General and University Hospital Alicante Spain; ^16^ Alicante Institute of Health and Biomedical Research (ISABIAL) Alicante Spain; ^17^ Biomedicine Institute of Valencia, Spanish Research Council (CSIC) Valencia Spain; ^18^ CIBER in Epidemiology and Public Health Spain; ^19^ Joint Research Unit “Infection and Public Health” FISABIO‐University of Valencia Valencia Spain; ^20^ Institute for Integrative Systems Biology (I2SysBio) CSIC‐University of Valencia Valencia Spain

**Keywords:** anti‐spike antibodies, breakthrough infection, Comirnaty® COVID‐19 vaccine, nursing home residents, SARS‐CoV‐2 Delta variant, spike‐reactive T cells

## Abstract

Severe acute respiratory syndrome coronavirus 2 (SARS‐CoV‐2) Delta variant breakthrough infections in nursing home residents following vaccination with Comirnaty® COVID‐19 vaccine were characterized. In total, 201 participants (median age, 87 years; range, 64–100; 133 female) from two nursing homes in the Valencian community (Spain) were included. SARS‐CoV‐2‐Spike (S) antibody responses were determined by a lateral flow immunocromatography (LFIC) assay and by quantitative electrochemiluminescent assay in LFIC‐negative participants. SARS‐CoV‐2‐S‐IFNγ T cells were enumerated by flow cytometry in 10 participants. Nasopharyngeal SARS‐CoV‐2 RNA loads were quantified by real‐time polymerase chain reaction assays. Vaccine breakthrough COVID‐19 due to the Delta variant occurred in 39 residents (median age, 87 years; range, 69–96; 31 female) at a median of 6.5 months after vaccination (nine requiring hospitalization). Breakthrough infections occurred at a higher rate *(p* < 0.0001) in residents who had not been previously infected with SARS‐CoV‐2 (naïve) (33/108; 18%) than in those with prior diagnosis of SARS‐CoV‐2 infection (experienced) (6/93; 6.4%), and were more likely (*p* < 0.0001) to develop in residents who tested negative by LFIC (20/49) at 3 months after vaccination as compared to their LFIC‐positive counterparts (19/142). Among LFIC‐negative residents, a trend towards lower plasma anti‐RBD antibody levels was noticed in those developing breakthrough infection (*p* = 0.16). SARS‐CoV‐2 RNA loads in nasopharyngeal specimens were lower in SARS‐CoV‐2‐experienced residents (*p* < 0.001) and in those testing positive by LFIC (*p* = 0.13). The frequency of SARS‐CoV‐2‐S‐reactive T cells at 3 months was similar in LFIC‐negative residents with (*n* = 7) or without (*n* = 3) breakthrough infection. Prior history of SARS‐CoV‐2 infection and detection of S‐reactive antibodies by LFIC at 3 months is associated with a lower risk of Delta‐variant breakthrough infection in nursing home residents at midterm after Comirnaty® COVID‐19 vaccination.

## 1 INTRODUCTION

Waning severe acute respiratory syndrome coronavirus 2 (SARS‐CoV‐2) messenger RNA (mRNA) vaccine protection against both asymptomatic infection and COVID‐19 has been documented in the general population, healthcare workers, and nursing home residents.^1^, ^2^, ^3^, ^4^, ^5^ This phenomenon is particularly worrisome in the latter collective given their inherent risk of developing severe forms of COVID‐19. In a recent study,^4^ the adjusted effectiveness of full mRNA vaccination against laboratory‐confirmed SARS‐CoV‐2 infection was reported to drop notably with the emergence of the Delta variant, although the net impact of this variant's involvement could not be discriminated from the potential link to the decline in vaccine‐induced immunity. The congregate nature of nursing home residences favors wide exposure of residents to SARS‐CoV‐2 following outbreak declaration, which potentially allows us to gauge the extent of protection afforded by postvaccination immunity against breakthrough infections. Here, we report on the relationship between SARS‐CoV‐2 immunity at 3 months after full administration of the Comirnaty® vaccine (two doses), as assessed in a previous study^6^ and subsequent occurrence of SARS‐CoV‐2 breakthrough infections due to the Delta variant in nursing home residents.

## 2 MATERIAL AND METHODS

### 2.1 Participants

Between June 2021 and October 2021, SARS‐CoV‐2 infection outbreak was documented in two Valencian Community (Spain) nursing homes (August 2021 in Nursing Home A [NHA], and June 2021 in NHB) involving in total 60 residents and 10 staff members (57 residents and 10 staff workers at NHA and 3 residents at NHB). Out of the total number of residents in these two nursing homes at the time of the outbreak declaration (*n* = 271), a total of 201 (median age, 87 years; range, 64–100; 133 female) had been evaluated for Comirnaty® COVID‐19 vaccine immunogenicity at a median of 3 months after full‐dose vaccination (two doses) in a previous study^6^ and were included in the current one. Thirty‐nine out of the 201 residents contracted breakthrough infection. The electronic Valencia Health System Integrated Databases (VID),^7^ was interrogated to ascertain the SARS‐CoV‐2 infection status of participants before the outbreak declaration. Residents were categorized as SARS‐CoV‐2 naïve or experienced according to the absence or presence, respectively of a real‐time polymerase chain reaction (RT‐PCR) positive registry in the above‐referred database. None of the residents included in the study either had a documented immunosuppression condition or was under immunosuppressive therapy at the time of vaccination. This study was undertaken as a primary task of the “Monitoring of antibody response following SARS‐CoV‐2 vaccination in nursing homes of the Valencian Community” program, carried out under the epidemiological surveillance competences of the Valencia Government Health Department (Law 16/2003/May 28 on Cohesion and Quality of the National Health System, and Law 10/2014/December 29 on Public Health of the Valencian Community). As such, informed consent, ethics approval, and publication of data were exempt from the approval of a research ethics committee. Personal data from nursing homes and residents were processed in accordance with European data protection regulations.

### 2.2 Virological documentation of SARS‐CoV‐2 vaccine‐breakthrough infections

As per local public health policy protocols, residents suspected of having COVID‐19 were tested within 24 h after symptoms onset for the presence of SARS‐CoV‐2 RNA in nasopharyngeal specimens (NP). This was carried out using either the Cobas® SARS‐CoV‐2 RT‐PCR assay (Roche Molecular Systems) for residents at NHA, or the Panther Fusion® SARS‐CoV‐2 assay (Hologic) for residents at NHB. RT‐PCR assays were run at the Microbiology Unit of Hospital Universitario de la Plana (Vila‐Real) or at the Microbiology Service of Consorci Hospital General Universitari de València. All asymptomatic residents were systematically tested by RT‐PCR within 48 h of the diagnosis of the index case and again twice or three times afterward after outbreak ending. SARS‐CoV‐2 viral loads in NP were estimated using the AMPLIRUN® TOTAL SARS‐CoV‐2 RNA Control (Vircell SA),^6^ and reported as copies/ml throughout the study. The SARS‐CoV‐2 variant involved in the outbreaks was determined by whole‐genome sequencing at the Foundation for the promotion of Health and Biomedical Research of the Valencian Community (FISABIO), as previously reported.^8^

### 2.3 Immunological testing strategy

We used a previously detailed immunological testing strategy^6^; briefly, a lateral flow immunochromatographic assay (LFIC) detecting anti‐SARS‐CoV‐2‐Spike(S) IgG and IgM antibodies (OnSite COVID‐19 IgG/IgM Rapid Test (CTK BIOTECH) in whole blood obtained by fingerstick was used as a front‐line assay for antibody testing. Qualitative results (positive vs. negative) were available from residents included in the current study. Participants returning negative results by LFIC immediately underwent venipuncture for further antibody testing by the Roche Elecsys® Anti‐SARS‐CoV‐2 S (Roche Diagnostics), which quantifies total antibodies against the S receptor‐binding domain (RBD). Specimens were assayed at the Microbiology Service of the Hospital Clínico Universitario of Valencia in singlets within 24 h of collection. A number of whole blood specimens were available from participants testing negative by LFIC for enumeration of SARS‐CoV‐2‐S‐reactive IFNγ‐producing‐CD8^+^ and CD4^+^ T cells at HCU by flow cytometry for ICS (BD Fastimmune, Becton Dickinson and Company‐Biosciences) as previously described.^6^

### 2.4 Statistical methods

Frequency comparisons for categorical variables were carried out using the Fisher exact test. Differences between medians were compared using the Mann–Whitney *U*‐test. The analyses were performed using SPSS version 20.0 (SPSS). Relative risks of SARS‐CoV‐2 breakthrough infections according to SARS‐CoV‐2 infection status before full vaccination and SARS‐CoV‐2 antibody detection by LFIC at 3 M following vaccination were calculated by Poisson regression analyses in R. Statistical significance was set at *p* < 0.05.

## 3 RESULTS

### 3.1 Clinical and virological features of SARS‐CoV‐2 breakthrough infection

Out of the 201 residents included in the study, 39 (median age, 87 years; range, 69–96; 31 females) contracted SARS‐CoV‐2 breakthrough infection (37 at NHA and 2 at NHB) due to the Delta variant, as documented by whole‐genome sequencing. Molecular diagnosis of SARS‐CoV‐2 infection was made at a median of 199 days (range, 83–217 days) after full vaccination with the Comirnaty® COVID‐19 vaccine. All 39 residents developed COVID‐19‐like symptoms, nine eventually required hospitalization, and three of these died. Six out of the 39 residents had record of SARS‐CoV‐2 infection occurring before outbreak declaration, specifically at a median of 89 days (range, 38–323 days) before full vaccination (two doses), due in all cases to the Wuhan‐Hu‐1 D614G variant.^7^ Overall, breakthrough infections occurred at a higher rate *(p* < 0.0001) in residents who had not been previously infected with SARS‐CoV‐2 (naïve) (33/108; 18%) than in those who had been (experienced) (6/93; 6.4%): specifically, the relative risk of contracting SARS‐CoV‐2 breakthrough infection was around two‐fold higher in the former than the latter (Table 1). All but one (8/9) hospitalized residents were SARS‐CoV‐2‐naïve.

**Table 1:** Relative risk of SARS‐CoV‐2 breakthrough infection in nursing home residents.

	RR	CI 95%
Age (>87 vs. ≤87)^a^	1.20	0.97–1.98
Sex (female vs. male)	1.26	1.03–1.54
SARS‐CoV‐2 infection status before outbreak declaration (naïve vs. experienced)^b^	1.82	1.47–2.26
LFIC test at 3 months after full vaccination (negative vs. positive)	2.86	1.82–4.50

Abbreviations: CI, confidence interval; LFIC, lateral flow immunochromatographic assay detecting IgG antibodies against SARS‐CoV‐2 Spike protein; RR, relative risk; SARS‐CoV‐2, severe acute respiratory syndrome coronavirus 2.

^a^ Median age of participants was set to establish comparison groups.

^b^ According to the electronic Valencia Health System Integrated Databases (VID).

**Figure 1:**
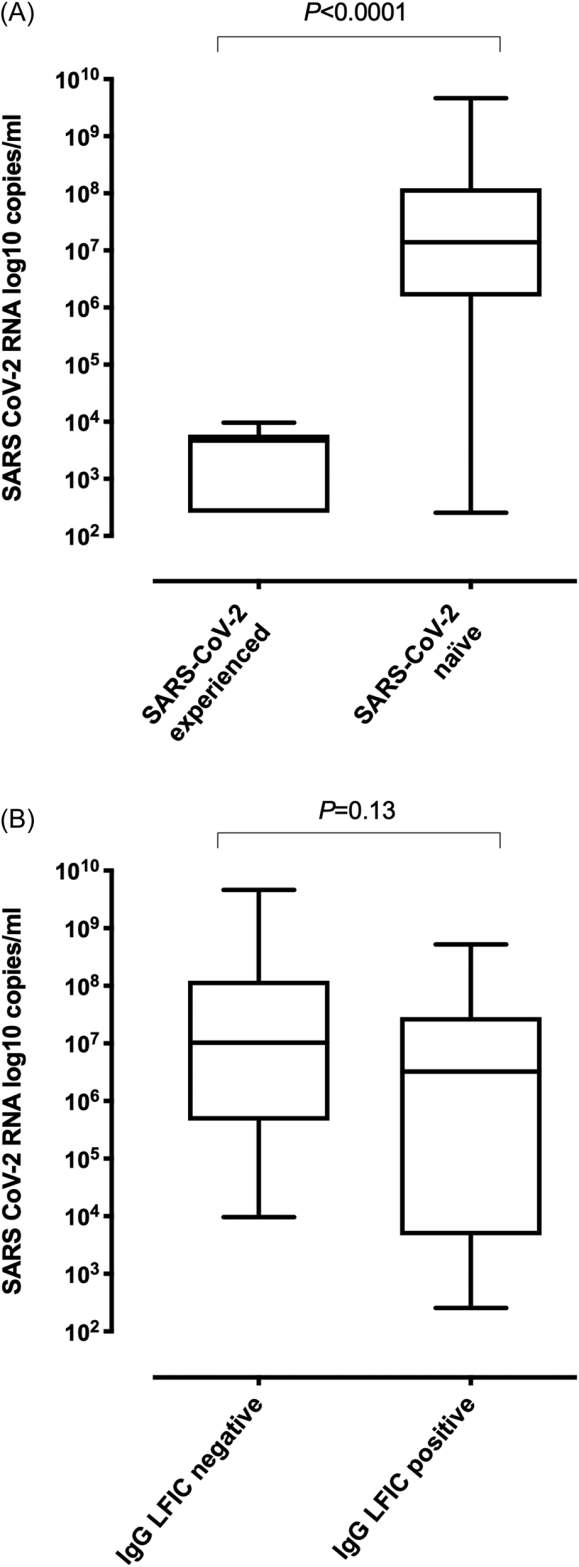
Box‐whisker plots representing SARS‐CoV‐2 RNA load in nasopharyngeal specimens as measured by commercial RT‐PCR assays calibrated to the AMPLIRUN® TOTAL SARS‐CoV‐2 RNA Control (Vircell SA), according to (A) SARS‐CoV‐2 infection status of participants with breakthrough infection before outbreak declaration (naïve; *n* = 33; experienced, *n* = 6) or (B) presence or absence of detectable anti‐S IgGs by a lateral flow immunochromatography (OnSite COVID‐19 IgG/IgM Rapid Test; CTK BIOTECH) in whole blood obtained by fingerstick at 3 months following complete vaccination with the Comirnaty® COVID‐19 vaccine in 39 nursing home residents. The *p* value is shown for comparisons. COVID‐19, coronavirus disease 2019; RT‐PCR, real‐time polymerase chain reaction; SARS‐CoV‐2, severe acute respiratory syndrome coronavirus 2

Overall, SARS‐CoV‐2 RNA load in NP at time of diagnosis was 6.8 log_10_ copies/ml (range, 2.4–9.7). As shown in Figure 1A, viral RNA load was significantly higher (*p* < 0.001) in SARS‐CoV‐2‐naïve than in experienced residents (median, 7.1 log_10_ copies/ml; range, 2.4–9.7 vs. median, 3.6 log_10_ copies/ml; range, 2.4–3.9). Interestingly, a trend towards (*p* = 0.06) a shorter time lapse between receipt of the second vaccine dose and diagnosis of SARS‐CoV‐2 breakthrough infection was noticed for SARS‐CoV‐2‐naïve compared to experienced participants (median, 173.5 days; range, 83–206 vs. median, 201 days; range, 115–217).

### 3.2 Occurrence of breakthrough infection according to postvaccination immune responses

Assessment of postvaccination immune responses were carried out at a median of 91 days (range, 21–103) before outbreak declaration and the results were overall reported in a previous study.^6^ Of the 201 residents, 49 returned negative results by LFIC, while the remaining 152 tested positive. Residents who developed SARS‐CoV‐2 breakthrough infection were more likely to test negative by LFIC (*p* < 0.0001) compared to their LFIC‐positive counterparts (Table 2). Testing negative by LFIC resulted in a 2.8‐fold increased risk of acquiring breakthrough infection (Table 1). Likewise, hospitalized patients tested negative by LFIC more frequently than nonhospitalized residents (*p* = 0.07) (Table 2). All three deceased residents returned negative LFIC results.

**Table 2:** SARS‐CoV‐2‐S‐reactive antibody and T‐cell responses in nursing home residents with or without breakthrough infections.

Immunological parameter	SARS‐CoV‐2 breakthrough infection	No SARS‐CoV‐2 breakthrough infection	*p* value	SARS‐CoV‐2 breakthrough infection requiring hospitalization	SARS‐CoV‐2 breakthrough infection not requiring hospitalization	*p* value
Positive IgG by LFIC (no. of residents)	19	133	<0.0001	2	17	0.075
Negative IgG by LFIC (no. of residents)	20	29		7	13	
Anti‐SARS‐CoV‐2‐RBD total antibodies in U (median; range)^a^	39 (0.4–949)	73.9 (0.4–2,099)	0.16	69.3; (49.3–2,099)	78.5; (0.4–368)	0.36
Frequency of SARS‐CoV‐2‐S‐reactive IFNγ‐producing‐CD8^+^ T cells (median %; range)^b^	0.13 (0.03–0.68)	0.15 (0–0.33)	0.73	NA	NA	
Frequency of SARS‐CoV‐2‐S‐reactive IFNγ‐producing‐CD4^+^ T cells (median%; range)^b^	0.48 (0.42–2.31)	1.22 (0.47–2.88)	0.30	NA	NA	

Abbreviations: IFNγ, interferon gamma; LFIC, lateral flow immunochromatography assay; NA, not applicable; RBD, receptor‐binding domain; SARS‐CoV‐2, severe acute respiratory syndrome coronavirus 2.

^a^ Performed in all 49 LFIC‐negative residents.

^b^ Performed in 10 LFIC‐negative residents (7 SARS‐CoV‐2 naïve).

**Figure 2:**
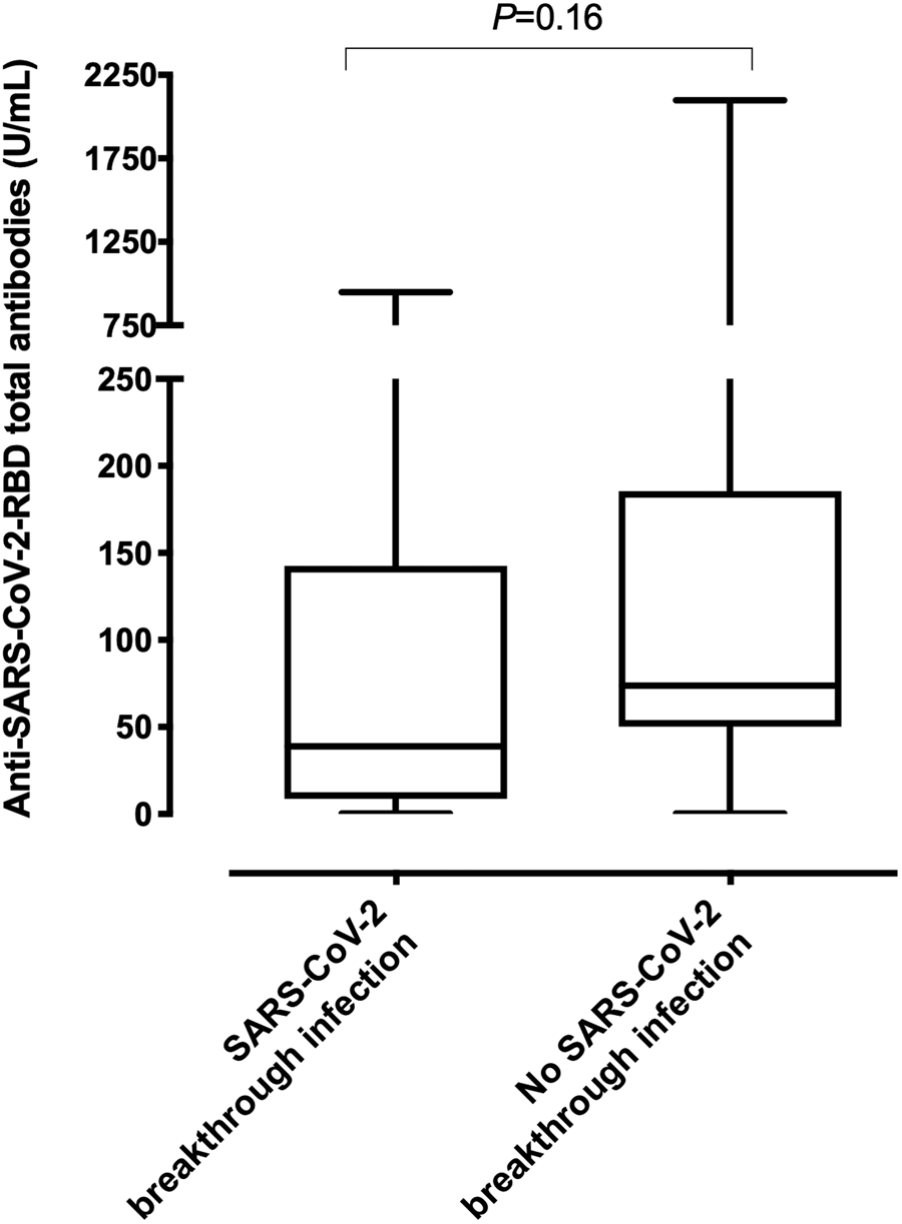
Box‐whisker plots depicting SARS‐CoV‐2 S receptor‐binding domain (RBD) total antibody levels as measured at 3 months following complete vaccination with the Comirnaty® COVID‐19 vaccine by the Roche Elecsys® Anti‐SARS‐CoV‐2 S (Roche Diagnostics, Pleasanton) in 49 nursing home residents testing negative by a lateral flow immunochromatography (OnSite COVID‐19 IgG/IgM Rapid Test; CTK BIOTECH) in whole blood obtained by fingerstick, either subsequently developing a breakthrough infection or not. The *p* value is shown for comparison. COVID‐19, coronavirus disease 2019; SARS‐CoV‐2, severe acute respiratory syndrome coronavirus 2

As per protocol, residents testing negative by LFIC (*n* = 49) were assessed for total anti‐RBD antibodies (Table 2). Antibodies were detectable in 19/20 and 24/29 residents with or without subsequent breakthrough infection (*p* = 0.20). As shown in Figure 2, a trend toward lower plasma anti‐RBD antibody levels was noticed in residents developing breakthrough infection (*p* = 0.16).

Among residents developing breakthrough infections, SARS‐CoV‐2 RNA loads in NP (Figure 1B) were slightly lower (*p* = 0.13) in those testing LFIC positive (median, 6.9 log_10_ copies/ml; range, 2.4–9.7), compared to participants testing LFIC negative (median, 6.5 log_10_ copies/ml; range (2.4–8.7).

Data on T‐cell immunity at 3 months were available from 10 LFIC‐negative/anti‐RBD‐positive residents, of whom three contracted vaccine breakthrough infection (none were hospitalized). The rate of detectable responses and the frequency of SARS‐CoV‐2‐S‐reactive‐IFNγ‐producing CD8^+^ and CD4^+^ T cells were similar across participants in the two comparison groups (Table 2).

## 4 DISCUSSION

The data reported herein strongly suggest that the strength of SARS‐CoV‐2‐S antibody responses persisting at 3 months after full vaccination with the Comirnaty® vaccine directly impacts the risk of Delta variant breakthrough infections occurring within 3–4 months afterward (median of 6.5 months after full vaccination) in elderly nursing home residents. Using an LFIC assay displaying a limit of detection for SARS‐CoV‐2‐S IgG of around 54.5 BAU/ml, as calibrated to the Roche anti‐RBD total antibody assay,^6^ we have shown that residents testing negative had an approximately threefold increased risk of contracting breakthrough infection compared with those returning positive results. Unfortunately, antibody reactivity grading in the LFIC assay,^6^ which could have allowed inference of plasma antibody levels (in a semi‐quantitative fashion), was not available for these residents, thus precluding any attempt to identify an antibody correlate of protection. Since residents maintaining detectable antibody responses by LFIC at 3 months were significantly more likely to have recovered from COVID‐19 before vaccination than SARS‐CoV‐2‐naïve subjects, not surprisingly the latter exhibited a higher risk of contracting breakthrough infection, in concurrence with previous observations.^9^ Moreover, among residents testing negative by LFIC, a trend towards lower anti‐RBD total antibody levels was noticed in those developing vaccine breakthrough infection. Likewise, we found that residents hospitalized because of COVID‐19 returned negative LFIC results at 3 months more frequently than their nonhospitalized counterparts. Moreover, all three residents who died tested negative by LFIC. To our best knowledge, ours is the first published data on the relationship between plasma/serum antibody levels following full vaccination with Comirnaty® COVID‐19 vaccine and the risk of breakthrough infection caused by the Delta variant (known to partially evade immune responses elicited by Wuhan‐Hu‐1‐based vaccines^10^) in elderly nursing home residents. Nonetheless, similar results to ours were found in healthcare staff, in whom serum neutralizing antibody titers measured 7–8 weeks after completion of the Oxford‐AstraZeneca vaccine schedule were inversely associated with risk of contracting SARS‐CoV‐2 Delta variant breakthrough infections.^11^

Elucidating the impact of vaccination on SARS‐CoV‐2 viral load in the upper respiratory tract in individuals with symptomatic or asymptomatic breakthrough infections is critically important from a public health perspective. Recent studies indicate that adaptive immune responses elicited by mRNA vaccines are initially effective in reducing viral loads of Delta breakthrough infections in the general population.^12^, ^13^; Our data seem to partly support this assumption in that overall SARS‐CoV‐2 RNA loads in NP were slightly higher but not significantly different in residents exhibiting poor antibody responses at a median of 3 months after full vaccination with Comirnaty® (those testing LFIC negative) than in those maintaining LFIC‐positive responses. Crucially, however, fully‐vaccinated SARS‐CoV‐2‐experienced residents exhibited significantly lower NP SARS‐CoV‐2 RNA loads than their naïve counterparts, suggesting that pre‐existing SARS‐CoV‐2 immunity (either boosted or not by vaccination) may dramatically hamper virus replication in the upper respiratory tract, even 6–7.5 months afterward.

Unfortunately, data on SARS‐CoV‐2‐S T‐cell immunity at 3 months were only available from a few participants, all of whom tested negative by LFIC and were thus presumed to have suboptimal vaccine responses.^6^ We found no differences across participants with or without subsequent breakthrough infection in either detection rate or frequency of SARS‐CoV‐2‐S‐reactive CD8^+^ and CD4^+^ T cells. Although these data must be interpreted with caution, they seem to suggest that vaccine breakthrough COVID‐19 may occur in the face of detectable SARS‐CoV‐2‐S T‐cell responses.

Several limitations of the current study must be acknowledged, the first being the modest number of breakthrough infections included. Second, we used an LFIC assay designed primarily to provide qualitative results instead of quantitative immunoassay for front‐line antibody testing. Indeed, anti‐RBD antibodies were only quantified in residents returning negative results by LFIC, although the extreme frailty of most participants made venipuncture unadvisable in the first place. Third, virus neutralization assays were not performed. Fourth, participant selection bias (testing only LFIC‐negative residents) may undermine the robustness of our conclusions regarding anti‐RBD antibody and S‐reactive T‐cell responses in participants developing or not breakthrough infection. Fifth, asymptomatic breakthrough infections could have been missed due to the policy of RT‐PCR testing in place during outbreaks; and finally, no data on peri‐outbreak antibody responses were available.

In summary, the data presented herein strongly suggest that for nursing home residents fully vaccinated with the Comirnaty® vaccine, pre‐existing immunity elicited by natural infection and persisting anti‐S antibody responses at 3 months after vaccination had a major impact on decreasing the relative risk of breakthrough Delta‐variant COVID‐19 at midterm and on limiting virus replication in the upper respiratory tract.

## AUTHOR CONTRIBUTIONS

Ignacio Torres and Eliseo Albert performed SARS‐CoV‐2 antibody and T‐cell assays at Hospital Clínico Universitario of Valencia. Concepción Gimeno and María Martínez‐Serrano, performed SARS‐CoV‐2 RT‐PCR assays at the Microbiology Service, of Consorcio Hospital General Universitario de Valencia. Helena Buj and Noelia Hernández performed SARS‐CoV‐2 RT‐PCR assays at the Laboratory Service of Hospital de la Plana. Juan B. Bellido‐Blasco, Raúl Moya‐Malo, Juan Carlos Gascó‐Laborda, Noemí Meseguer‐Ferrer, and Javier Ortíz‐Rambla were in charge of managing SARS‐CoV‐2 outbreaks at Nursing Home residence A. Ana Tornero and Josefa Soriano were in charge of managing SARS‐CoV‐2 outbreaks at Nursing Home residence B. Iñaki Comas and Fernando González‐Candelas carried out whole‐genome sequencing experiments. Javier S. Burgos, Salvador Peiró, Dolores Salas, Ramón Limón, Hermelinda Vanaclocha, José Sánchez‐Payá, and Javier Díez‐Domingo: conceptualization and data analysis. David Navarro: writing the original draft. All authors reviewed the original draft.

## CONFLICTS OF INTEREST

The authors declare no conflicts of interest.

## Data Availability

Data sharing is not applicable to this article as no new data were created or analyzed in this study.
